# Supplementary material for: Contingency management for tobacco smoking during opioid addiction treatment: a randomised pilot study
Source: BMJ Open. 2017 Sep 1;7(9):e017467. doi: 10.1136/bmjopen-2017-017467 (PMC5589007; doi:10.1136/bmjopen-2017-017467)
Supplement: Supplementary file 2 [file bmjopen-2017-017467supp002.pdf]

Figure. Example template of recommended content for the schedule of enrolment, interventions, and assessments.*

	Enrolment		Post-allocation					Post study	Follow-up
**TIMEPOINT****	*$-t_1$*	*Week 1*	*Week 2*	*Week 3*	*Week 4*	*Week 5*	*Week 6*		*6 Months*
**ENROLMENT:**									
**Eligibility screen**	X								
**Informed consent**	X								
Demographics collection	X								
**Allocation to condition**		X							
**INTERVENTIONS:**									
CM Smoking abstinence			◆—	—	—	—	—◆		
CM Clinic attendance			◆—	—	—	—	—◆		
**ASSESSMENTS:**									
**Numbers completing treatment**							X		
Demographics	X								
Breath CO			◆—	—	—	—	—◆		X
Point prevalence smoking			◆—	—	—	—	—◆		X
Opiate treatment information								X	
Illicit drug use								X	

*Recommended content can be displayed using various schematic formats. See SPIRIT 2013 Explanation and Elaboration for examples from protocols.
**List specific timepoints in this row.
