# Supplementary material for: Contingency management for tobacco smoking during opioid addiction treatment: a randomised pilot study
Source: BMJ Open. 2017 Sep 1;7(9):e017467. doi: 10.1136/bmjopen-2017-017467 (PMC5589007; doi:10.1136/bmjopen-2017-017467)
Supplement: Supplementary file 3 [file bmjopen-2017-017467supp003.pdf]

Appendix

Appendix 1 Participant Information Sheet

Study title: Addition of contingency management to stop smoking services for in-treatment opiate addicts: a randomised controlled pilot study

Participant Information Sheet

V1 14/03/2016
IRAS ID: 171709

South London and Maudsley NHS Foundation Trust

King's College London Institute of Psychiatry, Psychology & Neuroscience

Smoking Cessation Study

We invite you to take part in a research study

- Before deciding to take part in the study, it is important for you to understand why the research is being done and what taking part will involve
- Please take time to read the following information carefully and discuss it with friends and relatives if you wish
- You are free to decide whether or not to take part in this study. If you choose not to take part this will not affect the care that you receive at Lorraine Hewitt House or anywhere else
- If there is anything that is not clear or you would like more information, then please ask

Important things you need to know

- Tobacco smoking is very common amongst opiate and methadone users. This makes them likely to experience negative health effects.
- Stopping smoking is one of the best things you can do for your health. It can also reduce the discomfort resulting from opiate use treatment.
- Rewards are one way of helping people stop smoking. This is sometimes called contingency management.
- Contingency management has been shown to work well in changing lots of different behaviours. We want to see whether it could help opiate use patients to stop smoking.
- The aim of this study, is to see whether or not it would be possible to test this treatment in a larger trial
- If you take part in the study, you are free to withdraw from the study at any time, **without giving any reason**. If requested, any data that we have collected from you will be destroyed.
- We might ask you to fill out a small questionnaire if you do decide to withdraw from the study, to help us improve our interventions in the future. There is no obligation to complete this questionnaire though.

Requirements

In order to take part in the study you need to:

- Be in treatment for opiate addiction
- Smoke at least 10 cigarettes per day
- Be between 18 and 65 years old
- Must **NOT** be in treatment for any other drug addiction
- Must **NOT** be participating in any other research

What will taking part involve?

- This study is for people who want to stop smoking, and will attend the stop smoking clinic at Lorraine Hewitt house.
- You will need to come to Lorraine Hewitt House **once a week** on a Monday, Wednesday or Friday to attend the smoking clinic. You will have to do this for a total of 6 weeks.
- At the first study visit you will be asked questions about your age and work history etc. You will also be asked to plan to quit smoking for the following week.
- Every time you come into the clinic, you will have to blow into a machine that measures chemicals in your breath. This is how we know if you have been smoking or not.
- You will be put into one of two groups at random (e.g. by coin toss). In one group you can earn rewards for attending the stop smoking clinic and not smoking. In the other group you can earn rewards just for attending the clinic.
- Rewards will be 'Love2Shop' vouchers
- The amount of money that you earn each time you meet the criteria for that group (attending the stop smoking clinic and not smoking in one, and just attending the stop smoking clinic in the other) will start at £5 and will double each time you meet the criteria up to £40. The diagram below shows how much you will earn if you meet the criteria for reward for the duration of the study

	SSS Week/Visit Number						
	1	2	3	4	5	6	
	Intervention Week/Visit Number						
		1	2	3	4	5	**Total**
Reward Value	£0.00	£5.00	£10.00	£20.00	£40.00	£40.00	**£115.00**

- If you don't meet the criteria though you won't get paid for that visit to the clinic, and the amount that you get paid for the next time you do will start again at £5, and will increase each time like before.

Benefits of taking part

- You could earn up to £115 just by attending the stop smoking clinic and stopping smoking
- Taking part may help you to stop smoking for good. This will help improve your general health greatly
- By taking part, you will be helping us to better understand how we can help other people to stop smoking

Possible disadvantages of taking part

- Giving up smoking can result in a number of withdrawal symptoms that may cause discomfort. The behavioural support and nicotine replacement therapy that you will receive as part of the normal smoking cessation clinic is designed to help this.

Frequently asked questions and further information

- **What will happen if I don't want to carry on with the study?** If at any point during the study you decide that you no longer want to take part, you can withdraw without giving any reason. All you need to do is tell anyone at the clinic related with the study that this is the case, and you will immediately be withdrawn from the study. You can also request that all of the data collected from you be destroyed.
- **How will my information be kept confidential?** Any data stored about you will be anonymous, and will not contain any data that would allow you to be identified. All information recorded from you will be held on a secure computer system at King's College London, in an encrypted format that can only be accessed the research team involved with the study.
- **What will happen to the results of this study?** The results of this study will be used by the primary researcher Tom Ainscough as part of his doctoral thesis, will be written up as an academic paper to be published, and will help inform the design of future research.
- **What if I want to know the results of the study when it finishes?** If you want to be informed of the results of the study once it has finished, this information will be made freely available at Lorraine Hewitt House. Just ask at the reception.
- **Who is organising and funding this study?** The study is organised through the Institute of Psychology, Psychiatry and Neuroscience, King's College London and the South London and Maudsley NHS Trust, and is funded by the Medical Research Council (http://www.mrc.ac.uk/)
- **Who has reviewed this study?** The study design has been reviewed by both an NHS ethics committee and the Research and Development department of the Institute of Psychology, Psychiatry and Neuroscience, King's College London
- **Where can I find more information about research?** For more general information about research you can visit either http://www.invo.org.uk/ or www.testingtreatments.org
- **Who can I contact for more information about this study?** If you need any further information about the study, please contact Tom Ainscough by emailing thomas.ainscough@kcl.ac.uk or calling 020 7848 5727

Appendix 2 Demographic and Smoking behaviour questionnaire

**Basic Demographics**	
Participant ID	
Gender	Male, Female, Not reported
Pregnant	Yes, No, Not reported
Breastfeeding	Yes, No, Not reported
Eligible for free prescriptions?	Yes, No, Not reported
Ethnic Group	White British, White Irish, White Other, Mixed White & Black Caribbean, Mixed White & Black African, Mixed White & Asian, Mixed Other, Asia/Asian Brit – Indian, Asia/Asian Brit – Pakistani, Asia/Asian Brit – Bangladeshi, Asia/Asian Brit – Other, Black/Black Brit - Caribbean<br>Black/Black Brit – African, Black/Black Brit – Other, Chinese, Any other ethnic group
Employment Status	Full time student, Never Worked/Unemployed for over 1 year, Retired, Sick/Disabled/Unable to return to work, Home carer (unpaid), Managerial/Professional, Intermediate occupation (e.g. clerical worker), Routine & Manual occupation (e.g. electrician) Other
How did you hear about the service?	GP , Practice nurse, Pharmacist, Other Professional, NHS National smoking helpline, Internet, Family/Friends,

	Previous user of the service, Newspaper or magazine, TV, Poster/leaflet, Other

**Quitting**			
Quitting confidence	1 (Not at all) – 10 (Very)		
Quitting importance	1 (Not at all) – 10 (Very)		
Quitting Readiness	1 (Not at all) – 10 (Very)		
Tried to stop smoking before?	Yes / No	# Times:	
# weeks since last quit attempt			
Longest period of abstinence			
Have you tried NRT?	Yes / No	Types:	
		How long used for	
Ever tried Zyban/Champix?	Yes / No	How long used for	
Have you used other stop smoking aids?	Yes / No	Please Specify	

**Smoking Behaviour**	

What type of tobacco do you smoke?	Cigarettes, Roll-ups, Cigars, Oral
How many cig. Do you smoke per day?<br>(if hand rolled, how many ounces per<br>week - 0.5 oz is 12.5g, or 20 cigs)	
How soon after waking do you have your first cig.?	Less than 5 mins, 5-15 mins, 15-30 mins, 30-60 mins, 1-2 hours, More than 2 hours
How many years have you smoked?	
Age started smoking	
Live with a smoker?	

**Contact information**	
Mobile Phone	
Landline Number	
Email	-
Friend's contact details	-

Appendix 3 end of treatment satisfaction survey

Study title: Addition of contingency management to stop smoking services for in-treatment opiate addicts: a randomised controlled pilot study

South London and Maudsley NHS Foundation Trust | KING'S College LONDON | Institute of Psychiatry, Psychology & Neuroscience

Smoking Study Questionnaire

Participant ID: Date:

Please circle the ONE response you feel most appropriate:

1	**Would you recommend this service to other smokers who want to stop smoking?**	Yes	No	Unsure
2	**In the event that you started smoking again would you go back to the service for help with stopping smoking?**	Yes	No	Unsure
3	**If you returned to the service for help with stopping smoking in the future do you think that you would be welcomed back?**	Yes	No	Unsure
4	**When you contacted the service were you given an appointment date or told how long you would have to wait?**	Yes	No	Unsure
5	**Was the length of time you had to wait for your first appointment acceptable to you?**	Yes	No	Unsure
6	**Are the appointment times you were given convenient for you?**	Yes	No	Unsure
7	**Is the place where you go for your appointments convenient for you to get to?**	Yes	No	Unsure
8	**Was the information that you were given about the choice of medication helpful?**	Yes	No	Unsure
9	**Was it easy to get hold of your medicine once you had chosen which medication you were going to use for your stop smoking attempt?**	Yes	No	Unsure

Please circle the ONE response you feel most appropriate:

10	**Overall, how satisfied are you with the support you have received to stop smoking?**					
	Very Unsatisfied	Unsatisfied	Unsure	Satisfied	Very Satisfied	Not Applicable

11	**How satisfied are you with how supportive staff have been?**					
	Very Unsatisfied	Unsatisfied	Unsure	Satisfied	Very Satisfied	Not Applicable

12	**How helpful has the information and advice that staff have given to you during your appointment been?**					
	Very Unhelpful	Unhelpful	Unsure	Helpful	Very Helpful	Not Applicable

13	**How helpful has the written information that staff have given to you been?**					
	Very Unhelpful	Unhelpful	Unsure	Helpful	Very Helpful	Not Applicable

14	**Do you find having your carbon monoxide (CO) reading done at every visit helpful?**					
	Very Unhelpful	Unhelpful	Unsure	Helpful	Very Helpful	Not Applicable

Please tick ALL appropriate responses:

15	**Which of the following types of nicotine replacement therapy did you receive? (Please circle ALL that apply)**					
Nicotine patches	Nicotine gum	Nicotine lozenges	Inhalator	Mouth spray	Nasal spray	Oral Strips

16	**Which of the following types of nicotine replacement therapy did you find most useful? (Please circle ALL that apply)**					
Nicotine patches	Nicotine gum	Nicotine lozenges	Inhalator	Mouth spray	Nasal spray	Oral Strips

17	**Did you receive any vouchers during the intervention? If No please go to question 19**	
	Yes	No

18	If yes, how helpful were the vouchers in stopping smoking?					
	Very Unhelpful	Unhelpful	Unsure	Helpful	Very Helpful	Not Applicable

19	Would you have tried to quit smoking if there were no vouchers being offered?	
	Yes	No

20	Did you receive e-cigarettes during the study? If No, please go to question 22	
	Yes	No

21	If yes, how helpful were e-cigarettes for stopping smoking?					
	Very Unhelpful	Unhelpful	Unsure	Helpful	Very Helpful	Not Applicable

22	If you were to do the study again, what would be more likely to make you take part if you got:			
	Vouchers	Free E-cigarettes	Both	Other (please say):

23	Have you smoked since your last appointment with the service?			
	No, not a single puff	Yes, just a few puffs	Yes, 1-5 cigarettes	More Than 5 Cigarettes
