# Supplementary material for: Contingency management for tobacco smoking during opioid addiction treatment: a randomised pilot study
Source: BMJ Open. 2017 Sep 1;7(9):e017467. doi: 10.1136/bmjopen-2017-017467 (PMC5589007; doi:10.1136/bmjopen-2017-017467)
Supplement: Author's manuscript [file bmjopen-2017-017467.draft_revisions.pdf]

BMJ Open

BMJ Open is committed to open peer review. As part of this commitment we make the peer review history of every article we publish publicly available.

When an article is published we post the peer reviewers' comments and the authors' responses online. We also post the versions of the paper that were used during peer review. These are the versions that the peer review comments apply to.

The versions of the paper that follow are the versions that were submitted during the peer review process. They are not the versions of record or the final published versions. They should not be cited or distributed as the published version of this manuscript.

BMJ Open is an open access journal and the full, final, typeset and author-corrected version of record of the manuscript is available on our site with no access controls, subscription charges or pay-per-view fees (http://bmjopen.bmj.com).

If you have any questions on BMJ Open's open peer review process please email editorial.bmjopen@bmj.com

BMJ Open

Contingency management for tobacco smoking during opiate addiction treatment: a randomised pilot study

Journal:	*BMJ Open*
Manuscript ID	bmjopen-2017-017467
Article Type:	Protocol
Date Submitted by the Author:	25-Apr-2017

Complete List of Authors:	Ainscough, Tom; Institute of Psychiatry Psychology and Neuroscience Department of Addictions, ;<br>Brose, Leonie S.; King's College London, Addictions Department<br>Strang, John; Kings College London, Addictions<br>McNeill, Ann; King’s College London, UK Centre for Tobacco Control Studies, National Addiction Centre, Institute of Psychiatry

**Primary Subject Heading**:	Addiction
Secondary Subject Heading:	Addiction, Smoking and tobacco
Keywords:	Addicton, Tobacco, Smoking, Opiates, Contingency Management

SCHOLARONE™ Manuscripts

For peer review only

Contingency management for tobacco smoking during opiate addiction treatment: a randomised pilot study

Tom S Ainscough[1, 2], Leonie S Brose[1, 2], John Strang[2], Ann McNeill[1, 2]

[1] UK Centre for Tobacco and Alcohol Studies

[2]IoPPN, King's College London

Corresponding author: Tom S Ainscough, thomas.ainscough@kcl.ac.uk, 0207 8485727

Word Count: 3136

Number of Tables: 1

For peer review only

For peer review only - http://bmjopen.bmj.com/site/about/guidelines.xhtml

Abstract

Introduction: Smoking rates among individuals in treatment for opiate addiction are close to five times that of the general public. Moreover, drug addicted smokers have a premature mortality rate four times greater than drug addicted non-smokers. The aim of this pilot study is to investigate whether contingency management can be successfully added to evidence-based stop smoking treatment in individuals undergoing treatment for opiate addiction and assess preliminary evidence for its impact.

Methods and Analyses:

Participants: Forty tobacco smokers currently undergoing treatment for opiate addiction treatment.

Intervention: Escalating with reset contingency management as an adjunct to standard smoking cessation treatment. Financial incentives will be administered over a five-week period for either biochemically verified abstinence from smoking or attendance at the clinic. Participants will be randomised to conditions stratified on current levels of smoking (high or low).

Objectives and Analyses: To assess whether a CM intervention can be successfully added to standard stop smoking services treatment, in patients undergoing outpatient treatment for opiate addiction. This will be measured as the number of people completing the five weeks of the intervention.

Ethics and Dissemination: Ethics approval for the study was granted on the 16th of June 2016 by the London – city and east (reference 16/LO/0990) ethics committee. The pilot study was retrospectively registered on clincaltrials.gov in January 2017 (ID: NCT03015597). It is planned that the results of this study will be published in an academic journal.

For peer review only - http://bmjopen.bmj.com/site/about/guidelines.xhtml

Strengths and limitations:

Strengths:

- Extends an extensively tested evidence based intervention to a novel treatment population
- Implements a randomised controlled experimental design

Weaknesses:

- Due to constraints of the intervention blinding of both participants and treatment centre staff is not possible

Background

Tobacco smoking is the leading cause of premature death in the western world [1], currently killing six million people per year across the globe, predicted to rise to eight million people annually by 2030[2]. In England alone, smoking killed 74,000 people in 2014 [3]. Consequently, tobacco smoking places a large economic burden on both the National Health Service and the larger UK economy. It has been estimated that tobacco smoking costs the NHS approximately two billion pounds per year, with a total cost to the UK economy of approximately 13 billion pounds annually [4].

In 2016, smoking prevalence in the general UK population fell below 17% for the first time [5]. However, despite this encouraging downward trend, smoking prevalence amongst those in treatment for drug addiction remains high, with a prevalence of 88% recorded in the UK in 2013 [6], and little change observed in the 20 years from 1988 to 2008 [7]. Drug-addicted smokers also have a fourfold greater premature mortality rate than non-smokers [8]. This situation is further exacerbated by evidence showing that the efficacy of the standard stop smoking treatment currently used is nearly halved when an individual has used illicit drugs in the past 30 days [9]. There is therefore a great need for development of novel interventions for tobacco smoking for those in drug addiction treatment that can bolster the efficacy of current interventions. One of the highest rates of smoking prevalence in substance abuse treatment is observed in opiate addictions treatment, ranging between 84% and 98% [7, 10–13]. Moreover, those in treatment for opiate addiction report high rates of interest in stop smoking treatment [10, 11], making them an ideal population for the development of interventions for tobacco smoking in substance abuse treatment.

Contingency management (CM) is a behavioural intervention based on the principles of operant conditioning, whereby changes in behaviour are brought about by positively rewarding desired behaviours. CM has been shown to be an effective intervention for drug use during opiate addiction [14], and has been recommended for use in opiate addictions in the UK for some time [15]. Some studies show promising results for CM in smoking cessation during treatment for opiate addiction [16–19], however this remains under researched. Moreover, none of the currently published studies investigating this were carried out in the UK, or alongside standard stop smoking treatment.

For peer review only - http://bmjopen.bmj.com/site/about/guidelines.xhtml

The aim of the proposed pilot study is to assess whether a CM intervention can be successfully added to standard stop smoking services treatment, in patients undergoing outpatient treatment for opiate addiction.

Ethics

Risks to participants

There is no known risk associated with the CM behavioural intervention. Smoking cessation can precipitate a number of uncomfortable withdrawal symptoms. These will be attenuated by the stop smoking services treatment provided at the treatment centre, an evidence based treatment that includes nicotine replacement therapy, e-cigarettes and behavioural support. Any information recorded from participants will be anonymised using a participant ID number, the master sheet for which will be stored in a locked cabinet at the treatment centre. This ensures that no identifiable information will ever leave the treatment centre.

Vouchers rather than cash

Even though cash vouchers have been shown to be more effective than vouchers[20], the treatment centre where the pilot study is being carried out did not want participants to be paid in cash so as not be able to buy cigarettes, alcohol, or drugs. The "Love2Shop" vouchers that will be given to participants can be spent in a number of high street stores.

Informed consent

All participants will be given at least 24 hours after being given an information sheet to decide whether or not to take part, and will provide written consent, collected by the PI (TA). Participants will receive both the study intervention and standard stop smoking services treatment at no cost. The study received ethical approval from the London – city and east ethics committee on the 16th of June 2016 (reference 16/LO/0990).

Methods/Design

Objectives

Primary Objective: To investigate whether a CM intervention can be successfully added to standard stop smoking services treatment, in patients undergoing outpatient treatment for opiate addiction, in order to identify any elements that need changing before carrying out a full scale randomised controlled trial (RCT).

Secondary Objectives: To gather preliminary findings regarding the effects of the CM intervention on smoking in this group, and any possible effects the intervention may have on opiate addiction treatment outcomes.

For peer review only - http://bmjopen.bmj.com/site/about/guidelines.xhtml

Participants, recruitment, inclusion criteria and randomisation

As this is a pilot study, the primary outcome is not the efficacy of the study intervention. Consequently, the sample size has not been calculated to ascertain efficacy. Instead, the method outlined by Viechtbauer et al [21] for calculating the sample size based on the probability of any issues that may arise has been used. A sample size of 40 using the above rationale is powerful enough to provide over 90% certainty of detecting any issues that occur with a probability of over 5%.

The study therefore aims to recruit 40 patients, all undergoing current treatment for opiate addiction and who smoke ten or more cigarettes a day. Participants will be recruited from the study site, a drug addiction treatment centre, either through self-referrals in response to advertisements shown in the treatment centre, or referrals from treatment centre staff. Participants are eligible for inclusion if they want to quit smoking (complete abstinence), are between 18 and 65 years old, undergoing pharmacological treatment for opiate addiction, smoke a minimum of ten cigarettes per day, and provide informed consent. Participants will be ineligible for inclusion in the study if they exhibit insufficient English skills to understand study protocols, are currently undergoing treatment for other drugs of abuse or if taking part in other research. Pregnant women will not be excluded.

Participants will be randomised into either experimental (CM for abstinence) or control (CM for attendance) conditions when recruited into the trial. Randomisation will be performed by the PI, using the service provided by the company ‘sealed envelope ltd.’[22], and will be performed using random permuted blocks within strata. Randomisation will be stratified based on participants’ current smoking frequency (between 10 and 20 per day, and more than 20 per day [6]).

Study design

A two-arm randomised controlled pilot study with six-month follow-up. The intervention will be provided as an adjunct to the standard smoking cessation treatment provided at the treatment centre, with CM rewards available during weeks 2 to 5 of the smoking cessation treatment. The study will be conducted in compliance with the principles of the Declaration of Helsinki [23], the principles of Good Clinical Practice, and all applicable regulatory requirements. The main elements of the study are outlined in the flow chart (Figure 1).

Standard Treatment

The standard smoking cessation treatment provided at the treatment centre follows the treatment program set out by the National Centre for Smoking Cessation and Training (NCSCT) [24] and The National Institute for Health and Care Excellence (NICE) guidelines for smoking cessation [25]. This treatment combines manualised behavioural support to stop smoking with nicotine replacement therapy (NRT), and takes place over six weeks with one

For peer review only - http://bmjopen.bmj.com/site/about/guidelines.xhtml

session per week. In the context of drug addiction treatment, service users are sometimes offered treatment over a slightly longer period of time. In the first meeting, the service user's readiness and ability to quit is assessed, information for the remainder of the treatment program is given and a quit date for the next week is set. For the remaining five weeks, clients attend the clinic to receive behavioural support and have their abstinence biochemically verified. In the study clinic, NRT is available free of charge to all individuals engaged with smoking cessation treatment, in the form of nicotine patches, gum, inhalators, mouth or oral spray, and oral strips. At the time of the study, the clinic is also additionally offering (on a trial basis) e-cigarettes, which have a nicotine content of 18mg/ml. These e-cigarettes are disposable and securely sealed, initially designed for use in high-security environments like prisons [26]. The smoking cessation treatment provided at the treatment centre does not include treatment with bupropion.

During the six weeks of treatment, service users are given a week's supply of NRT or e-cigarettes at a time. At the end of the six weeks, service users are given a two-week supply of NRT or e-cigarettes before exiting the treatment. The type of NRT received is decided by clients with guidance from the cessation worker, and can constitute a single form of NRT or a combination of different types. Clients' breath carbon monoxide (CO) levels are measured using a Bedfont piCO+ Smokerlyzer breath CO monitor. Measurements are taken at the initial visit and at each subsequent visit over the next five weeks, to biochemically verify self-reported abstinence from smoking (CO<10ppm [27]). NRT and e-cigarette use is recorded throughout treatment.

CM Intervention

The CM intervention will run as an adjunct to the normal smoking cessation treatment, and follows an escalating with reset schedule. In escalating with reset CM, rewards increase in a set increment value for each successive verified display of the desired behaviour. When the desired behaviour is not observed, no reward is given, and the reward value for the next verified display of the desired behaviour is reset to that of the initial reward. Reward values then begin to rise again in the same way as before. The CM intervention will run for five weeks in total, starting in week two of the standard stop smoking services treatment and ending in week six (Figure 2). Randomisation will be performed after collection of demographics following taking of consent. Participants will be rewarded for smoking abstinence in the experimental condition, or for attending the smoking cessation clinic in the control condition. Smoking abstinence will be defined as a breath CO reading of <10ppm, and attendance will be defined as attending the smoking cessation treatment at the clinic that week. After each smoking cessation treatment session, the cessation worker will fill out a slip that records each participant's individual participant number and his or her breath CO for that day. The cessation worker will give these slips to the PI who will sit in an adjacent room and will administer rewards where appropriate. All participant data will be recorded using participant numbers ensuring that no identifiable data leaves the clinic, and will be stored in an encrypted file, separate to a sheet matching participant names to IDs which will be kept in a locked office at the treatment centre. Due to the nature of the CM intervention, it is not possible to blind participants to treatment allocation. Cessation workers will not be made aware of treatment allocation; however, they cannot be considered to be blinded to treatment allocation as it is possible that clients may discuss this with them.

For peer review only - http://bmjopen.bmj.com/site/about/guidelines.xhtml

Reward values will be the same in both conditions and begin at £5, doubling each time the incentivised behaviour is recorded to a maximum of £40. All rewards will be given as "Love2Shop" vouchers. Over the course of the whole intervention, participants will be able to earn a maximum of £115 (Table 1). At the end of the CM intervention participants will be asked to complete a client satisfaction and well-being survey, previously used to assess client satisfaction of stop smoking services treatment [28].

Table 1. Reward schedule

Smoking Cessation Treatment Week Number	1	2	3	4	5	6
CM Week Number		1	2	3	4	5
Reward Value	£0.00	£5.00	£10.00	£20.00	£40.00	£40.00

Reward schedule for a participant that remains abstinent and/or attends all smoking cessation treatment meetings (dependent on condition) for the duration of the intervention. Maximum total reward: £115

Measures

Outcome measures

The primary outcome will be assessed by recording the number of participants completing the five weeks of the intervention in each condition. Success will be defined as 60% or more of participants completing treatment.

The secondary objectives of the study are to gather preliminary findings regarding the effects of the CM intervention on smoking in this group, and any possible effects the intervention may have on opiate addiction treatment outcomes. Smoking abstinence will be recorded as point prevalence and biochemically verified with abstinence defined as a breath CO reading of under 10ppm [27]. Participant medical records will be accessed to ascertain participants' opiate addiction treatment, including drug types (methadone, Subutex etc.) and dosage as well as illicit drug use throughout the period of the trial.

Follow-up measures

At the six month follow up (see below for follow-up procedures), the following measures will be recorded:

Point prevalence smoking abstinence: Self-reported smoking abstinence for 7 days before follow-up and exhaled air CO <10ppm [27].

Continuous abstinence: Self-reported smoking abstinence since end of treatment and exhaled air CO<10ppm. Participants smoking five or fewer cigarettes during the six-month follow-up will be considered self-reported quitters [27].

Illicit drug use, collected at the end of the study from participants' medical records.

All those lost to follow-up will be treated as smoking [27].

Other measures

At the first stop smoking treatment session, a number of demographic and smoking behaviour variables will be recorded. The collection form for this information is shown in Appendix 1. As many contact details as possible will also be taken for the participants in order to increase

For peer review only - http://bmjopen.bmj.com/site/about/guidelines.xhtml

the probability of participants being able to be followed up. This will include the details of relevant friends and family members.

Follow up Procedures

Six months after their set quit date, participants will be contacted by the PI to ascertain their self-reported smoking status. In order to test the optimal follow up method, participants will be pseudo-randomised by recruitment order to be contacted by text and phone call, or email and phone call. All participants will also be asked to return to the clinic in order to have their breath CO levels tested to verify abstinence. Once this is done, participants will have completed their participation in the study. Participants will receive a £10 voucher for completing the follow up procedure.

Planned analysis

As the primary objective of the intervention does not entail any hypothesis testing, the only statistics reported for this will be descriptive, namely means and standard deviations for the number of participants retained at the end of treatment in each condition. Baseline demographics will be compared between conditions using t-tests for continuous and chi square for categorical data to ensure that any differences in these are not driving any potential differences in retention.

For the secondary objectives: differences between the groups in smoking cessation will be investigated using chi square test; data for opiate use will be compared between conditions using t-tests and chi square tests dependent on data and any questionnaire data will be reported using descriptive statistics. All statistics will be performed as two tailed tests using an alpha value of 0.05.

Discussion

The addition of contingent incentives to standard evidence-based smoking cessation treatment in opiate addiction clients will be an innovative approach, having never been attempted before in the UK. If feasible, the intervention will be easily disseminated, and has the potential to be an effective intervention for smoking in this client group. Pilot studies are an imperative step in the development of complex interventions, and form the first step on the road to full scale RCT and potentially implementation [29, 30]. If successful, this program paves the way for the development of a full scale RCT of CM for smoking in opiate addiction treatment which would include an economic evaluation, and potential trials for smokers in other drug addiction treatment.

For peer review only - http://bmjopen.bmj.com/site/about/guidelines.xhtml

List of abbreviations

CM – Contingency management, RCT – Randomised Controlled Trial, CO – Carbon Monoxide.

Declarations

Ethics approval and consent to participate

Ethics approval for the study was granted on the 16th of June 2016 by the London – city and east (reference 16/LO/0990) ethics committee.

Sponsor contact details:

Trial Sponsor: King's College London and South London and Maudsley NHS Foundation Trust

Contact name: Jennifer Liebscher

Address: Research & Development Office, Box P005, Institute of Psychiatry, Psychology & Neuroscience, King's College London, Denmark Hill Campus, 16 De Crespigny Park, London SE5 8AF

Telephone: 020 7848 0251

Email: jennifer.liebscher@kcl.ac.uk

Consent for publication

Not applicable

Availability of data and material

The datasets used and/or analysed during the current study available from the corresponding author on reasonable request**.**

Competing interests

The authors declare that they have no competing interests

Funding

This work was funded as part of TA's PhD studentship by the Medical Research Council and the Institute of Psychiatry, Psychology and Neuroscience (MRC/IoP Excellence Studentship). LB is funded by a Cancer Research UK (CRUK)/ BUPA Foundation Cancer Prevention Fellowship (C52999/ A19748). LB and AM are members of the UK Centre for Tobacco and Alcohol Studies, a UK Clinical Research Collaboration Public Health Research: Centre of Excellence. Funding from the Medical Research Council, British Heart Foundation, Cancer Research UK, Economic and Social Research Council and the National Institute for Health Research under the auspices of the UK Clinical Research Collaboration is gratefully acknowledged 35 (MR/K/K023195/1). JS has contributed to UK guidelines which include consideration of the potential role of contingency management in the management of addiction problems (NICE, 2007; chaired by JS), and JS also chaired the broader-scope pan-

For peer review only - http://bmjopen.bmj.com/site/about/guidelines.xhtml

UK working group preparing the 2007 and 2017 editions of the 'Orange Book' ('Guidelines on the Management of Drug Misuse & Dependence') for the UK Departments of Health, providing guidance on management and treatment of drug dependence and misuse, which include guidance on possible inclusion of contingency management. JS's institution has received support and funding from the Department of Health (England) and National Treatment Agency (England), and JS and JS's institution have provided funded consultancy advice on possible novel addiction treatments, products and formulations to a range of pharmaceutical companies but these do not have any connection to the intervention being investigated in this paper. JS's employer (King's College London) has registered intellectual property on a novel buccal naloxone with which JS is involved, and JS has been named in a patent registration by a pharmaceutical company as inventor of a potential novel concentrated nasal spray, but these do not have any connection to the work being reported in this paper. A fuller account of JS's interests is at http://www.kcl.ac.uk/ioppn/depts/addictions/people/hod.aspx. JS is supported by the National Institute for Health Research (NIHR) Biomedical Research Centre for Mental Health at South London and Maudsley NHS Foundation Trust and King's College London and is a NIHR Senior Investigator.

Neither the funding bodies nor study sponsors had any role in study design; collection, management, analysis, and interpretation of data; writing of the report; and the decision to submit the report for publication.

Authors' contributions

TA was responsible for the design of the study with input from AM, LB and JS. TA is responsible for the recruitment of participants and for the collection, and analysis of participant data with input from AM and LB.

Acknowledgements

Not applicable

For peer review only

References

1. World Health Organization. Global Health Observatory data repository. Tobacco use Data by country. 2015. http://apps.who.int/gho/data/view.main.1805?lang=en.

2. World Health Organization. WHO report on the global tobacco epidemic, 2011: warning about the dangers of tobacco. Geneva: World Health Organization; 2011.

3. Office for National Statistics. Statistics on Smoking, England 2016. 2016.

4. Action on Smoking and Health. The economics of tobacco. 2015.

5. Office for National Statistics. Annual Population Survey, April 2014 - March 2015. 2016.

6. Cookson C, Strang J, Ratschen E, Sutherland G, Finch E, McNeill A. Smoking and its treatment in addiction services: Clients' and staff behaviour and attitudes. BMC Health Serv Res. 2014;14:304.

7. Guydish J, Passalacqua E, Tajima B, Chan M, Chun J, Bostrom A. Smoking prevalence in addiction treatment: a review. Nicotine Tob Res. 2011;13:401–11.

8. Hser YI, Mccarthy WJ, Anglin MD. Tobacco use as a distal predictor of mortality among long-term narcotics addicts. Prev Med (Baltim). 1994;23:61–9.

9. Stapleton JA, Keaney F, Sutherland G. Illicit drug use as a predictor of smoking cessation treatment outcome. Nicotine Tob Res. 2009;11:685–9.

10. Bowman J, Wiggers J, Colyvas K, Wye P, Walsh RA, Bartlem K. Smoking cessation among Australian methadone clients: Prevalence, characteristics and a need for action. Drug Alcohol Rev. 2012;31:507–13.

11. Clemmey P, Brooner R, Chutuape MA, Kidorf M, Stitzer M. Smoking habits and attitudes in a methadone maintenance treatment population. Drug Alcohol Depend. 1997;44:123–32.

12. Pajusco B, Chiamulera C, Quaglio G, Moro L, Casari R, Amen G, et al. Tobacco Addiction and Smoking Status in Heroin Addicts under Methadone vs. Buprenorphine Therapy. Int J Environ Res Public Health. 2012;9:932–42.

13. Tacke U, Wolff K, Finch E, Strang J. The effect of tobacco smoking on subjective symptoms of inadequacy ("not holding") of methadone dose among opiate addicts in methadone maintenance treatment. Addict Biol. 2001;6:137–45.

14. Griffith JD, Rowan-Szal GA, Roark RR, Simpson DDD. Contingency management in outpatient methadone treatment: a meta-analysis. Drug Alcohol Depend. 2000;58:55–66.

15. Pilling S, Strang J, Gerada C. NICE GUIDELINES: Psychosocial interventions and opioid detoxification for drug misuse: summary of NICE guidance. Br Med J. 2007;335:203–5.

16. Tuten M, Fitzsimons H, Chisolm MS, Nuzzo P a, Jones HE. Contingent incentives reduce cigarette smoking among pregnant, methadone-maintained women: results of an initial feasibility and efficacy randomized clinical trial. Addiction. 2012;107:1868–77.

17. Shoptaw S, Rotheram-Fuller E, Yang X, Frosch D, Nahom D, Jarvik ME, et al. Smoking cessation in methadone maintenance. Addiction. 2002;97:1317–28.

For peer review only - http://bmjopen.bmj.com/site/about/guidelines.xhtml

18. Dunn, K. E, Sigmon, S. C, Thomas, C. S, Heil, S. H, Higgins, S. T, Dunn KE, et al. Voucher-based contingent reinforcement of smoking abstinence among methadone-maintained patients: a pilot study. J Appl Behav Anal. 2008;41:527–38.

19. Dunn KE, Sigmon SC, Reimann EF, Badger GJ, Heil SH, Higgins ST. A Contingency-Management Intervention to Promote Initial Smoking Cessation Among Opioid-Maintained Patients. Exp Clin Psychopharmacol. 2010;18:37–50.

20. Topp L, Islam MM, Day CA. Relative efficacy of cash versus vouchers in engaging opioid substitution treatment clients in survey-based research. Journal of Medical Ethics. 2013;39:253–6.

21. Viechtbauer W, Smits L, Kotz D, Budé L, Spigt M, Serroyen J, et al. A simple formula for the calculation of sample size in pilot studies. J Clin Epidemiol. 2015;68:1375–9.

22. Sealed Envelope Ltd. Simple randomisation service. 2016. https://www.sealedenvelope.com/simple-randomiser/v1/.

23. World Medical Association Declaration of Helsinki: ethical principles for medical research involving human subjects. JAMA. 2013;310:2191–4.

24. McEwen A. National Centre for Smoking Cessation and Training (NCSCT) Standard Treatment Programme. Second Edition. 2014.

25. National Institute for Health and Care Excellence. Smoking: supporting people to stop. 2012. http://www.nice.org.uk/guidance/qs43/resources/smoking-supporting-people-to-stop-2098665030085. Accessed 29 Jan 2016.

26. E-Burn. Product information - e-burn. http://e-burn.com/product-information/. Accessed 23 Feb 2017.

27. West R, Hajek P, Stead L, Stapleton J. Outcome criteria in smoking cessation trials: proposal for a common standard. Addiction. 2005;100:299–303.

28. Dobbie F, Hiscock R, Leonardi-Bee J, Murray S, Shahab L, Aveyard P, et al. Evaluating Long-term Outcomes of NHS Stop Smoking Services (ELONS): a prospective cohort study. Health Technol Assess. 2015;19:1–156.

29. Thabane L, Ma J, Chu R, Cheng J, Ismaila A, Rios LP, et al. A tutorial on pilot studies: the what, why and how. BMC Med Res Methodol. 2010;10:1.

30. Senn B, Kirsch M, Sanz CC, Karlou C, Tulus K, De Leeuw J, et al. Developing and evaluating complex interventions: the new Medical Research Council guidance. Studies. 2013;59:587–92.

For peer review only - http://bmjopen.bmj.com/site/about/guidelines.xhtml

Appendix

Appendix 1 Demographic and Smoking behaviour questionnaire

**Basic Demographics**	
Participant ID	
Gender	
Pregnant	
Breastfeeding	
Eligible for free prescriptions?	
Ethnic Group	
Employment Status	
How did you hear about the service?	

**Quitting**				
Quitting confidence				
Quitting importance				
Quitting Readiness				
Tried to stop smoking before?			# Times:	
# weeks since last quit attempt				
Longest period of abstinence				
Have you tried NRT?			Types:	
			How long used for	
Ever tried Zyban/Champix?			How long used for	

For peer review only

Have you used other stop smoking aids?			Please Specify	

**Smoking Behaviour**	
What type of tobacco do you smoke?	
How many cig. Do you smoke per day?<br>(if hand rolled, how many ounces per<br>week - 0.5 oz is 12.5g, or 20 cigs)	
How soon after waking do you have your first cig.?	
How many years have you smoked?	
Age started smoking	
Live with a smoker?	

**Contact information**	
Mobile Phone	
Landline Number	
Email	-
Friend's contact details	-

For peer review only

Figure**.** Example template of recommended content for the schedule of enrolment, interventions, and assessments.*

	Enrolment		Post-allocation					Post study	Follow-up
**TIMEPOINT****	*$-t_1$*	*Week 1*	*Week 2*	*Week 3*	*Week 4*	*Week 5*	*Week 6*		*6 Months*
**ENROLMENT:**									
**Eligibility screen**	X								
**Informed consent**	X								
Demographics collection	X								
**Allocation to condition**		X							
**INTERVENTIONS:**									
CM Smoking abstinence			◆——	——	——	——	——◆		
CM Clinic attendance			◆——	——	——	——	——◆		
**ASSESSMENTS:**									
**Numbers completing treatment**							X		
Demographics	X								
Breath CO			◆——	——	——	——	——◆		X
Point prevalence smoking			◆——	——	——	——	——◆		X
Opiate treatment information								X	
Illicit drug use								X	

*Recommended content can be displayed using various schematic formats. See SPIRIT 2013 Explanation and Elaboration for examples from protocols.
**List specific timepoints in this row.

For peer review only

SPIRIT

STANDARD PROTOCOL ITEMS: RECOMMENDATIONS FOR INTERVENTIONAL TRIALS

SPIRIT 2013 Checklist: Recommended items to address in a clinical trial protocol and related documents*

Section/item	Item No	Description	Addressed on page number
**Administrative information**			
Title	1	Descriptive title identifying the study design, population, interventions, and, if applicable, trial acronym	______1_______
Trial registration	2a	Trial identifier and registry name. If not yet registered, name of intended registry	______1_______
	2b	All items from the World Health Organization Trial Registration Data Set	_____N/A______
Protocol version	3	Date and version identifier	______________
Funding	4	Sources and types of financial, material, and other support	______9______
Roles and responsibilities	5a	Names, affiliations, and roles of protocol contributors	_____1, 9______
	5b	Name and contact information for the trial sponsor	______9_______
	5c	Role of study sponsor and funders, if any, in study design; collection, management, analysis, and interpretation of data; writing of the report; and the decision to submit the report for publication, including whether they will have ultimate authority over any of these activities	______10_______
	5d	Composition, roles, and responsibilities of the coordinating centre, steering committee, endpoint adjudication committee, data management team, and other individuals or groups overseeing the trial, if applicable (see Item 21a for data monitoring committee)	______N/A______

For peer review only

For peer review only - http://bmjopen.bmj.com/site/about/guidelines.xhtml

Introduction

Background and rationale	6a	Description of research question and justification for undertaking the trial, including summary of relevant studies (published and unpublished) examining benefits and harms for each intervention	_____1,2_______
	6b	Explanation for choice of comparators	____ 1-4____
Objectives	7	Specific objectives or hypotheses	_______3______
Trial design	8	Description of trial design including type of trial (eg, parallel group, crossover, factorial, single group), allocation ratio, and framework (eg, superiority, equivalence, noninferiority, exploratory)	____3-7_______

Methods: Participants, interventions, and outcomes

Study setting	9	Description of study settings (eg, community clinic, academic hospital) and list of countries where data will be collected. Reference to where list of study sites can be obtained	______5-6______
Eligibility criteria	10	Inclusion and exclusion criteria for participants. If applicable, eligibility criteria for study centres and individuals who will perform the interventions (eg, surgeons, psychotherapists)	_______3_______
Interventions	11a	Interventions for each group with sufficient detail to allow replication, including how and when they will be administered	______5-7______
	11b	Criteria for discontinuing or modifying allocated interventions for a given trial participant (eg, drug dose change in response to harms, participant request, or improving/worsening disease)	_______2_______
	11c	Strategies to improve adherence to intervention protocols, and any procedures for monitoring adherence (eg, drug tablet return, laboratory tests)	_______N/A______
	11d	Relevant concomitant care and interventions that are permitted or prohibited during the trial	_______6______
Outcomes	12	Primary, secondary, and other outcomes, including the specific measurement variable (eg, systolic blood pressure), analysis metric (eg, change from baseline, final value, time to event), method of aggregation (eg, median, proportion), and time point for each outcome. Explanation of the clinical relevance of chosen efficacy and harm outcomes is strongly recommended	_______6_______
Participant timeline	13	Time schedule of enrolment, interventions (including any run-ins and washouts), assessments, and visits for participants. A schematic diagram is highly recommended (see Figure)	_______5_______

For peer review only - http://bmjopen.bmj.com/site/about/guidelines.xhtml

Sample size	14	Estimated number of participants needed to achieve study objectives and how it was determined, including clinical and statistical assumptions supporting any sample size calculations	______5_______
Recruitment	15	Strategies for achieving adequate participant enrolment to reach target sample size	______5_______
**Methods: Assignment of interventions (for controlled trials)**			
Allocation:			
Sequence generation	16a	Method of generating the allocation sequence (eg, computer-generated random numbers), and list of any factors for stratification. To reduce predictability of a random sequence, details of any planned restriction (eg, blocking) should be provided in a separate document that is unavailable to those who enrol participants or assign interventions	______4_______
Allocation concealment mechanism	16b	Mechanism of implementing the allocation sequence (eg, central telephone; sequentially numbered, opaque, sealed envelopes), describing any steps to conceal the sequence until interventions are assigned	______4_______
Implementation	16c	Who will generate the allocation sequence, who will enrol participants, and who will assign participants to interventions	______4_______
Blinding (masking)	17a	Who will be blinded after assignment to interventions (eg, trial participants, care providers, outcome assessors, data analysts), and how	_____ 4______
	17b	If blinded, circumstances under which unblinding is permissible, and procedure for revealing a participant's allocated intervention during the trial	_____N/A______
**Methods: Data collection, management, and analysis**			
Data collection methods	18a	Plans for assessment and collection of outcome, baseline, and other trial data, including any related processes to promote data quality (eg, duplicate measurements, training of assessors) and a description of study instruments (eg, questionnaires, laboratory tests) along with their reliability and validity, if known. Reference to where data collection forms can be found, if not in the protocol	______6-7______
	18b	Plans to promote participant retention and complete follow-up, including list of any outcome data to be collected for participants who discontinue or deviate from intervention protocols	______8______

For peer review only - http://bmjopen.bmj.com/site/about/guidelines.xhtml

Data management	19	Plans for data entry, coding, security, and storage, including any related processes to promote data quality (eg, double data entry; range checks for data values). Reference to where details of data management procedures can be found, if not in the protocol	______N/A______
Statistical methods	20a	Statistical methods for analysing primary and secondary outcomes. Reference to where other details of the statistical analysis plan can be found, if not in the protocol	______8_______
	20b	Methods for any additional analyses (eg, subgroup and adjusted analyses)	______N/A______
	20c	Definition of analysis population relating to protocol non-adherence (eg, as randomised analysis), and any statistical methods to handle missing data (eg, multiple imputation)	_______6__ _____
**Methods: Monitoring**			
Data monitoring	21a	Composition of data monitoring committee (DMC); summary of its role and reporting structure; statement of whether it is independent from the sponsor and competing interests; and reference to where further details about its charter can be found, if not in the protocol. Alternatively, an explanation of why a DMC is not needed	______N/A _____
	21b	Description of any interim analyses and stopping guidelines, including who will have access to these interim results and make the final decision to terminate the trial	______N/A _____
Harms	22	Plans for collecting, assessing, reporting, and managing solicited and spontaneously reported adverse events and other unintended effects of trial interventions or trial conduct	______N/A _____
Auditing	23	Frequency and procedures for auditing trial conduct, if any, and whether the process will be independent from investigators and the sponsor	_____________
**Ethics and dissemination**			
Research ethics approval	24	Plans for seeking research ethics committee/institutional review board (REC/IRB) approval	______9_______
Protocol amendments	25	Plans for communicating important protocol modifications (eg, changes to eligibility criteria, outcomes, analyses) to relevant parties (eg, investigators, REC/IRBs, trial participants, trial registries, journals, regulators)	______N/A______

For peer review only - http://bmjopen.bmj.com/site/about/guidelines.xhtml

Consent or assent	26a	Who will obtain informed consent or assent from potential trial participants or authorised surrogates, and how (see Item 32)	______3______
	26b	Additional consent provisions for collection and use of participant data and biological specimens in ancillary studies, if applicable	_______N/A ______
Confidentiality	27	How personal information about potential and enrolled participants will be collected, shared, and maintained in order to protect confidentiality before, during, and after the trial	_______7________
Declaration of interests	28	Financial and other competing interests for principal investigators for the overall trial and each study site	______9-10______
Access to data	29	Statement of who will have access to the final trial dataset, and disclosure of contractual agreements that limit such access for investigators	_______9________
Ancillary and post-trial care	30	Provisions, if any, for ancillary and post-trial care, and for compensation to those who suffer harm from trial participation	_______N/A______
Dissemination policy	31a	Plans for investigators and sponsor to communicate trial results to participants, healthcare professionals, the public, and other relevant groups (eg, via publication, reporting in results databases, or other data sharing arrangements), including any publication restrictions	______________
	31b	Authorship eligibility guidelines and any intended use of professional writers	_______N/A______
	31c	Plans, if any, for granting public access to the full protocol, participant-level dataset, and statistical code	_____________
**Appendices**			
Informed consent materials	32	Model consent form and other related documentation given to participants and authorised surrogates	______________
Biological specimens	33	Plans for collection, laboratory evaluation, and storage of biological specimens for genetic or molecular analysis in the current trial and for future use in ancillary studies, if applicable	_______N/A_______

*It is strongly recommended that this checklist be read in conjunction with the SPIRIT 2013 Explanation & Elaboration for important clarification on the items. Amendments to the protocol should be tracked and dated. The SPIRIT checklist is copyrighted by the SPIRIT Group under the Creative Commons "Attribution-NonCommercial-NoDerivs 3.0 Unported" license.

For peer review only - http://bmjopen.bmj.com/site/about/guidelines.xhtml

Contingency management for tobacco smoking during opioid addiction treatment: a randomised pilot study

Journal:	*BMJ Open*
Manuscript ID	bmjopen-2017-017467.R1
Article Type:	Protocol
Date Submitted by the Author:	12-Jun-2017
Complete List of Authors:	Ainscough, Tom; Institute of Psychiatry Psychology and Neuroscience Department of Addictions, ;<br>Brose, Leonie; King's College London, Addictions Department<br>Strang, John; Kings College London, Addictions<br>McNeill, Ann; King's College London, UK Centre for Tobacco Control Studies, National Addiction Centre, Institute of Psychiatry
**Primary Subject Heading**:	Addiction
Secondary Subject Heading:	Addiction, Smoking and tobacco
Keywords:	Addicton, Tobacco, Smoking, Opiates, Contingency Management

For peer review only - http://bmjopen.bmj.com/site/about/guidelines.xhtml

Contingency management for tobacco smoking during opioid addiction treatment: a randomised pilot study

Tom S Ainscough[1, 2], Leonie S Brose[1, 2], John Strang[2], Ann McNeill[1, 2]

[1] UK Centre for Tobacco and Alcohol Studies

[2]IoPPN, King's College London

Corresponding author: Tom S Ainscough, thomas.ainscough@kcl.ac.uk, 0207 8485727

Word Count: 3136

Number of Tables: 1

For peer review only

Abstract

Introduction: Smoking rates among individuals in treatment for opioid addiction are close to five times that of the general public. Moreover, drug addicted smokers have a premature mortality rate four times greater than drug addicted non-smokers. The aim of this pilot study is to investigate whether contingency management can be successfully added to evidence-based stop smoking treatment in individuals undergoing treatment for opioid addiction and assess preliminary evidence for its impact.

Methods and Analyses:

Participants: Forty tobacco smokers currently undergoing treatment for opioid addiction treatment.

Intervention: Escalating with reset contingency management as an adjunct to standard smoking cessation treatment. Financial incentives will be administered over a five-week period for either biochemically verified abstinence from smoking or attendance at the clinic. Participants will be randomised to conditions stratified on current levels of smoking (high or low).

Objectives and Analyses: To assess whether a CM intervention can be successfully added to standard stop smoking services treatment, in patients undergoing outpatient treatment for opioid addiction. This will be measured as the number of people completing the five weeks of the intervention.

Ethics and Dissemination: Ethics approval for the study was granted on the 16th of June 2016 by the London – city and east (reference 16/LO/0990) ethics committee. The pilot study was retrospectively registered on clincaltrials.gov in January 2017 (ID: NCT03015597). A SPIRIT checklist and figure are available for this protocol (see supplementary materials). It is planned that the results of this study will be published in an academic journal.

For peer review only - http://bmjopen.bmj.com/site/about/guidelines.xhtml

Strengths and limitations:

Strengths:

- Extends an extensively tested evidence based intervention to a novel treatment population
- Implements a randomised controlled experimental design

Weaknesses:

- Due to constraints of the intervention blinding of both participants and treatment centre staff is not possible

Background

Tobacco smoking is the leading cause of premature death in the western world [1], currently killing six million people per year across the globe, predicted to rise to eight million people annually by 2030[2]. In England alone, smoking killed 74,000 people in 2014 [3]. Consequently, tobacco smoking places a large economic burden on both the National Health Service and the larger UK economy. It has been estimated that tobacco smoking costs the NHS approximately two billion pounds per year, with a total cost to the UK economy of approximately 13 billion pounds annually [4].

In 2016, smoking prevalence in the general UK population fell below 17% for the first time [5]. However, despite this encouraging downward trend, smoking prevalence amongst those in treatment for drug addiction remains high, with a prevalence of 88% recorded in the UK in 2013 [6], and little change observed in the 20 years from 1988 to 2008 [7]. Drug-addicted smokers also have a fourfold greater premature mortality rate than non-smokers [8]. This situation is further exacerbated by evidence showing that the efficacy of the standard stop smoking treatment currently used is nearly halved when an individual has used illicit drugs in the past 30 days [9]. There is therefore a great need for development of novel interventions for tobacco smoking for those in drug addiction treatment that can bolster the efficacy of current interventions. One of the highest rates of smoking prevalence in substance abuse treatment is observed in opioid addictions treatment, ranging between 84% and 98% [7,10–13]. Moreover, those in treatment for opioid addiction report high rates of interest in stop smoking treatment [10,11], making them an ideal population for the development of interventions for tobacco smoking in substance abuse treatment.

Contingency management (CM) is a behavioural intervention based on the principles of operant conditioning, whereby changes in behaviour are brought about by positively rewarding desired behaviours. CM has been shown to be an effective intervention for drug use during opioid addiction [14], and has been recommended for use in opioid addictions in the UK for some time [15]. Some studies show promising results for CM in smoking cessation during treatment for opioid addiction [16–20], however this remains under researched. Moreover, none of the currently published studies investigating this were carried out in the UK, or alongside standard stop smoking treatment.

For peer review only - http://bmjopen.bmj.com/site/about/guidelines.xhtml

The aim of the proposed pilot study is to assess whether a CM intervention can be successfully added to standard stop smoking services treatment, in patients undergoing outpatient treatment for opioid addiction.

Ethics

Risks to participants

There is no known risk associated with the CM behavioural intervention. Smoking cessation can precipitate a number of uncomfortable withdrawal symptoms. These will be attenuated by the stop smoking services treatment provided at the treatment centre, an evidence based treatment that includes nicotine replacement therapy, e-cigarettes and behavioural support. Any information recorded from participants will be anonymised using a participant ID number, the master sheet for which will be stored in a locked cabinet at the treatment centre. This ensures that no identifiable information will ever leave the treatment centre.

Vouchers rather than cash

The treatment centre where the pilot study is being carried out did not want participants to be paid in cash so as not be able to buy cigarettes, alcohol, or drugs. The “Love2Shop” vouchers used as an alternative can be spent in a number of high street stores. Although cash vouchers have been shown to be more effective than vouchers in some case [21], other research has shown cash and monetary vouchers to be of equal efficacy [22,23]. The use of monetary vouchers therefore should not negatively impinge on the efficacy of the current intervention. Participants will receive both the study intervention and standard stop smoking services treatment at no cost.

Methods/Design

Objectives

Primary Objective: To investigate whether a CM intervention can be successfully added to standard stop smoking services treatment, in patients undergoing outpatient treatment for opioid addiction, in order to identify any elements that need changing before carrying out a full scale randomised controlled trial (RCT).

Secondary Objectives: To gather preliminary findings regarding the effects of the CM intervention on smoking in this group, and any possible effects the intervention may have on opioid addiction treatment outcomes.

Participants, recruitment, inclusion criteria and randomisation

As this is a pilot study, the primary outcome is not the efficacy of the study intervention. Consequently, the sample size has not been calculated to ascertain efficacy. Instead, the method outlined by Viechtbauer et al [24] for calculating the sample size based on the

For peer review only - http://bmjopen.bmj.com/site/about/guidelines.xhtml

probability of any issues that may arise has been used. A sample size of 40 using the above rationale is powerful enough to provide over 90% certainty of detecting any issues that occur with a probability of over 5%.

The study therefore aims to recruit 40 patients, all undergoing current treatment for opioid addiction and who smoke ten or more cigarettes a day. Participants will be recruited from the study site, a drug addiction treatment centre, either through self-referrals in response to advertisements shown in the treatment centre, or referrals from treatment centre staff. Participants are eligible for inclusion if they want to quit smoking (complete abstinence), are between 18 and 65 years old, undergoing pharmacological treatment for opioid addiction, smoke a minimum of ten cigarettes per day, and provide informed consent. Use of smoking cessation medication is not a criterion for exclusion. Participants will be ineligible for inclusion in the study if they exhibit insufficient English skills to understand study protocols, are currently undergoing treatment for other drugs of abuse or if taking part in other research. Pregnant women will not be excluded.

Participants will be randomised into either experimental (CM for abstinence) or control (CM for attendance) conditions when recruited into the trial. Randomisation will be performed by the PI, using the service provided by the company 'sealed envelope ltd.'[25], and will be performed using random permuted blocks within strata. Randomisation will be stratified based on participants' current smoking frequency (between 10 and 20 per day, and more than 20 per day [6]). All participants will be given at least 24 hours after being given an information sheet to decide whether or not to take part, and will provide written consent, collected by the PI (TA).

Study design

A two-arm randomised controlled pilot study with six-month follow-up. The intervention will be provided as an adjunct to the standard smoking cessation treatment provided at the treatment centre, with CM rewards available during weeks 2 to 5 of the smoking cessation treatment. The study will be conducted in compliance with the principles of the Declaration of Helsinki [26], the principles of Good Clinical Practice, and all applicable regulatory requirements.

Opioid Treatment

As part of the standard opioid treatment programme the clinic offers both behavioural and pharmacological treatments. Pharmacological treatments include methadone, buprenorphine and in some cases a combination of buprenorphine and naloxone; each of these progresses from a daily supervised dose, to a daily unsupervised pickup to a weekly unsupervised pickup. All medication prescriptions are reviewed every six months. Clients are also allocated a key worker with whom they meet in person every two weeks to discuss their treatment, and who can refer them to a number of different behavioural support programs. These include psychological therapies or group therapy for their drug use, or a number of other services for issues related to their drug use such as needle exchanges, blood-borne virus testing and domestic violence support. In the past, the clinic has implemented CM interventions as part

For peer review only - http://bmjopen.bmj.com/site/about/guidelines.xhtml

of other research projects, however CM has never been implemented as part of the standard opioid treatment program.

Standard Treatment

Prior to the initiation of the current study, the smoking clinic had not operated for several months; smoking cessation training was re-administered to clinic staff and the smoking cessation treatment re-launched prior to the start of the trial. The treatment runs at the same time each week, on a Monday afternoon from 2-4 PM. The standard smoking cessation treatment provided at the treatment centre follows the treatment program set out by the National Centre for Smoking Cessation and Training (NCSCT) [27] and The National Institute for Health and Care Excellence (NICE) guidelines for smoking cessation [28]. This treatment combines manualised behavioural support to stop smoking with nicotine replacement therapy (NRT), and takes place over six weeks with one session per week. In the context of drug addiction treatment, service users are sometimes offered treatment over a slightly longer period of time. In the first meeting, the service user's readiness and ability to quit is assessed, information for the remainder of the treatment program is given and a quit date for the next week is set. For the remaining five weeks, clients attend the clinic to receive behavioural support and have their abstinence biochemically verified. In the study clinic, NRT is available free of charge to all individuals engaged with smoking cessation treatment, in the form of nicotine patches, gum, inhalators, mouth or oral spray, and oral strips. At the time of the study, the clinic is also additionally offering (on a trial basis) e-cigarettes, which have a nicotine content of 18mg/ml. These e-cigarettes are disposable and securely sealed, initially designed for use in high-security environments like prisons [29]. The smoking cessation treatment provided at the treatment centre does not include treatment with bupropion.

During the six weeks of treatment, service users are given a week's supply of NRT or e-cigarettes at a time. At the end of the six weeks, service users are given a two-week supply of NRT or e-cigarettes before exiting the treatment. The type of NRT received is decided by clients with guidance from the cessation worker, and can constitute a single form of NRT or a combination of different types. Clients' breath carbon monoxide (CO) levels are measured using a Bedfont piCO+ Smokerlyzer breath CO monitor. Measurements are taken at the initial visit and at each subsequent visit over the next five weeks, to biochemically verify self-reported abstinence from smoking (CO<10ppm [30]). NRT and e-cigarette use is recorded throughout treatment. Participants are made aware of these procedures in the participant information sheet that they are given prior to signing consent to the study (see appendix 2).

CM Intervention

The CM intervention will run as an adjunct to the normal smoking cessation treatment, and follows an escalating with reset schedule. In escalating with reset CM, rewards increase in a set increment value for each successive verified display of the desired behaviour. When the desired behaviour is not observed, no reward is given, and the reward value for the next verified display of the desired behaviour is reset to that of the initial reward. Reward values

For peer review only - http://bmjopen.bmj.com/site/about/guidelines.xhtml

then begin to rise again in the same way as before. The CM intervention will run for five weeks in total, starting in week two of the standard stop smoking services treatment and ending in week six (Table 1). Randomisation will be performed after collection of demographics following taking of consent. Participants will be rewarded for smoking abstinence in the experimental condition, or for attending the smoking cessation clinic in the control condition. Smoking abstinence will be defined as a breath CO reading of <10ppm, and attendance will be defined as attending the smoking cessation treatment at the clinic that week. After each smoking cessation treatment session, the cessation worker will fill out a slip that records each participant's individual participant number and his or her breath CO for that day. The cessation worker will give these slips to the PI who will sit in an adjacent room and will administer rewards where appropriate. All participant data will be recorded using participant numbers ensuring that no identifiable data leaves the clinic, and will be stored in an encrypted file, separate to a sheet matching participant names to IDs which will be kept in a locked office at the treatment centre. Due to the nature of the CM intervention, it is not possible to blind participants to treatment allocation. Cessation workers will not be made aware of treatment allocation; however, they cannot be considered to be blinded to treatment allocation as it is possible that clients may discuss this with them.

Reward values will be the same in both conditions and begin at £5, doubling each time the incentivised behaviour is recorded to a maximum of £40. All rewards will be given as "Love2Shop" vouchers. Over the course of the whole intervention, participants will be able to earn a maximum of £115 (Table 1). At the end of the CM intervention participants will be asked to complete a client satisfaction and well-being survey, previously used to assess client satisfaction of stop smoking services treatment [31].

Table 1. Reward schedule

Smoking Cessation Treatment Week Number	1	2	3	4	5	6
CM Week Number		1	2	3	4	5
Reward Value	£0.00	£5.00	£10.00	£20.00	£40.00	£40.00

Reward schedule for a participant that remains abstinent and/or attends all smoking cessation treatment meetings (dependent on condition) for the duration of the intervention. Maximum total reward: £115

Measures

Outcome measures

The primary outcome will be assessed by recording the number of participants completing the five weeks of the intervention in each condition. Success will be defined as 60% or more of participants completing treatment.

The secondary objectives of the study are to gather preliminary findings regarding the effects of the CM intervention on smoking in this group, and any possible effects the intervention may have on opioid addiction treatment outcomes. Smoking abstinence will be recorded as point prevalence and biochemically verified with abstinence defined as a breath CO reading of under 10ppm [27]. Participants were informed that smoking cannabis would increase CO levels.

For peer review only - http://bmjopen.bmj.com/site/about/guidelines.xhtml

Participant medical records will be accessed after completion of the intervention to ascertain participants' opioid addiction treatment, including treatment adherence, drug types (methadone, Subutex etc.), dosage and schedule (daily supervised pickup, weekly pickup etc.) as well as illicit drug use throughout the period of the trial.

Follow-up measures

At the six month follow up (see below for follow-up procedures), the following measures will be recorded:

Point prevalence smoking abstinence: Self-reported smoking abstinence for 7 days before follow-up and exhaled air CO <10ppm [30].

Continuous abstinence: Self-reported smoking abstinence since end of treatment and exhaled air CO<10ppm. Participants smoking five or fewer cigarettes during the six-month follow-up will be considered self-reported quitters [30].

Illicit drug use, collected at the end of the study from participants' medical records.

All those lost to follow-up will be treated as smoking [30].

Other measures

At the first stop smoking treatment session, a number of demographic and smoking behaviour variables will be recorded. The collection form for this information is shown in Appendix 1. As many contact details as possible will also be taken for the participants in order to increase the probability of participants being able to be followed up. This will include the details of relevant friends and family members. Participants will also complete a satisfaction questionnaire on the last day of their participation in the trial, which will assess a number of satisfaction criteria including the value of incentives received (see appendix 3).

Follow up Procedures

Six months after their set quit date, participants will be contacted by the PI to ascertain their self-reported smoking status. The main purpose of this follow-up is to ascertain whether participants can be successfully followed up for six months, and no group differences are expected to be found between the different conditions. In order to test the optimal follow up method, participants will be pseudo-randomised by recruitment order to be contacted by text and phone call, or email and phone call. All participants will also be asked to return to the clinic in order to have their breath CO levels tested to verify abstinence. Once this is done, participants will have completed their participation in the study. Participants will receive a £10 voucher for completing the follow up procedure.

Planned analysis

As the primary objective of the intervention does not entail any hypothesis testing, the only statistics reported for this will be descriptive, namely means and standard deviations for the number of participants retained at the end of treatment in each condition. Baseline demographics will be compared between conditions using t-tests for continuous and chi square for categorical data to ensure that any differences in these are not driving any potential differences in retention.

For peer review only - http://bmjopen.bmj.com/site/about/guidelines.xhtml

For the secondary objectives: differences between the groups in smoking cessation will be investigated using chi square test; differences between conditions on opioid use and opioid treatment during the intervention will be compared using t-tests and chi square tests dependent on data and any questionnaire data will be reported using descriptive statistics. All statistics will be performed as two tailed tests using an alpha value of 0.05.

Discussion

The addition of contingent incentives to standard evidence-based smoking cessation treatment in opiate addiction clients will be an innovative approach, having never been attempted before in the UK.

The current trial has a number of limitations that should be improved upon in future studies. Firstly, the value and frequency of rewards in the current study are comparatively lower than those of previous trials and should therefore be increased to encourage the cessation. The use of breath CO only in measuring abstinence is not the most rigorous method available of testing abstinence, due to the relatively short period of time it takes for breath CO levels to return to levels considered as those of a non-smoker. Urine cotinine levels provide a more rigorous measure of abstinence, however are confounded by the use of NRT therefore necessitating the measurement of anabasine instead. The measurement of both cotinine and anabasine were beyond the scope of the current intervention. Furthermore, provision of incentives to participants in the attendance group should come before breath CO levels are measures to avoid the risk of these participants thinking their incentives are linked to CO levels.

The intervention has a number of potential strengths however. If feasible, the intervention will be easily disseminated, and has the potential to be an effective intervention for smoking in this client group. Pilot studies are an imperative step in the development of complex interventions, and form the first step on the road to full scale RCT and potentially implementation [32,33]. If successful, this program paves the way for the development of a full scale RCT of CM for smoking in opiate addiction treatment which would include an economic evaluation, and potential trials for smokers in other drug addiction treatment.

List of abbreviations

CM – Contingency management, RCT – Randomised Controlled Trial, CO – Carbon Monoxide.

Declarations

Ethics approval and consent to participate

Ethics approval for the study was granted on the 16th of June 2016 by the London – city and east (reference 16/LO/0990) ethics committee.

Sponsor contact details:

For peer review only - http://bmjopen.bmj.com/site/about/guidelines.xhtml

Trial Sponsor: King's College London and South London and Maudsley NHS Foundation Trust

Contact name: Jennifer Liebscher

Address: Research & Development Office, Box P005, Institute of Psychiatry, Psychology & Neuroscience, King's College London, Denmark Hill Campus, 16 De Crespigny Park, London SE5 8AF

Telephone: 020 7848 0251

Email: jennifer.liebscher@kcl.ac.uk

Consent for publication

Not applicable

Availability of data and material

The datasets used and/or analysed during the current study available from the corresponding author on reasonable request.

Competing interests

JS has contributed to UK guidelines which include consideration of the potential role of contingency management in the management of addiction problems (NICE, 2007; chaired by JS), and JS also chaired the broader-scope pan-UK working group preparing the 2007 and 2017 editions of the 'Orange Book' ('Guidelines on the Management of Drug Misuse & Dependence') for the UK Departments of Health, providing guidance on management and treatment of drug dependence and misuse, which include guidance on possible inclusion of contingency management. JS's institution has received support and funding from the Department of Health (England) and National Treatment Agency (England), and JS and JS's institution have provided funded consultancy advice on possible novel addiction treatments, products and formulations to a range of pharmaceutical companies but these do not have any connection to the intervention being investigated in this paper. JS's employer (King's College London) has registered intellectual property on a novel buccal naloxone with which JS is involved, and JS has been named in a patent registration by a pharmaceutical company as inventor of a potential novel concentrated nasal spray, but these do not have any connection to the work being reported in this paper. A fuller account of JS's interests is at http://www.kcl.ac.uk/ioppn/depts/addictions/people/hod.aspx. JS is supported by the National Institute for Health Research (NIHR) Biomedical Research Centre for Mental Health at South London and Maudsley NHS Foundation Trust and King's College London and is a NIHR Senior Investigator.

Funding

This work was funded as part of TA's PhD studentship by the Medical Research Council and the Institute of Psychiatry, Psychology and Neuroscience (MRC/IoP Excellence Studentship). LB is funded by a Cancer Research UK (CRUK)/ BUPA Foundation Cancer Prevention Fellowship (C52999/ A19748). LB and AM are members of the UK Centre for Tobacco and Alcohol Studies, a UK Clinical Research Collaboration Public Health Research: Centre of Excellence. Funding from the Medical Research Council, British Heart Foundation, Cancer Research UK, Economic and Social Research Council and the National Institute for Health

For peer review only - http://bmjopen.bmj.com/site/about/guidelines.xhtml

Research under the auspices of the UK Clinical Research Collaboration is gratefully acknowledged 35 (MR/K/K023195/1).

Neither the funding bodies nor study sponsors had any role in study design; collection, management, analysis, and interpretation of data; writing of the report; and the decision to submit the report for publication.

Authors' contributions

TA was responsible for the design of the study with input from AM, LB and JS. TA is responsible for the recruitment of participants and for the collection, and analysis of participant data with input from AM and LB.

Acknowledgements

Not applicable

For peer review only

For peer review only - http://bmjopen.bmj.com/site/about/guidelines.xhtml

References

1 World Health Organization. Global Health Observatory data repository. Tob. use Data by Ctry. 2015.http://apps.who.int/gho/data/view.main.1805?lang=en

2 World Health Organization. *WHO report on the global tobacco epidemic, 2011: warning about the dangers of tobacco.* Geneva: : World Health Organization 2011.

3 Office for National Statistics. Statistics on Smoking, England 2016. 2016. doi:10.1038/1811181a0

4 Action on Smoking and Health. The economics of tobacco. 2015.

5 Office for National Statistics. *Annual Population Survey, April 2014 - March 2015.* 2016. doi:http://dx.doi.org/10.5255/UKDA-SN-7742-3

6 Cookson C, Strang J, Ratschen E, *et al.* Smoking and its treatment in addiction services: Clients' and staff behaviour and attitudes. *BMC Health Serv Res* 2014;**14**:304. doi:10.1186/1472-6963-14-304

7 Guydish J, Passalacqua E, Tajima B, *et al.* Smoking prevalence in addiction treatment: a review. *Nicotine Tob Res* 2011;**13**:401–11. doi:10.1093/ntr/ntr048

8 Hser YI, Mccarthy WJ, Anglin MD. Tobacco use as a distal predictor of mortality among long-term narcotics addicts. *Prev Med (Baltim)* 1994;**23**:61–9. doi:10.1006/pmed.1994.1009

9 Stapleton JA, Keaney F, Sutherland G. Illicit drug use as a predictor of smoking cessation treatment outcome. *Nicotine Tob Res* 2009;**11**:685–9. doi:10.1093/ntr/ntp050

10 Bowman J, Wiggers J, Colyvas K, *et al.* Smoking cessation among Australian methadone clients: Prevalence, characteristics and a need for action. *Drug Alcohol Rev* 2012;**31**:507–13. doi:10.1111/j.1465-3362.2011.00408.x

11 Clemmey P, Brooner R, Chutuape MA, *et al.* Smoking habits and attitudes in a methadone maintenance treatment population. *Drug Alcohol Depend* 1997;**44**:123–32. doi:10.1016/S0376-8716(96)01331-2

12 Pajusco B, Chiamulera C, Quaglio G, *et al.* Tobacco Addiction and Smoking Status in Heroin Addicts under Methadone vs. Buprenorphine Therapy. *Int J Environ Res Public Health* 2012;**9**:932–42. doi:10.3390/ijerph9030932

13 Tacke U, Wolff K, Finch E, *et al.* The effect of tobacco smoking on subjective symptoms of inadequacy of methadone dose among opiate addicts in methadone maintenance treatment. *Addict Biol* 2001;**6**:137–45. doi:10.1080/13556210020040217

14 Griffith JD, Rowan-Szal GA, Roark RR, *et al.* Contingency management in outpatient methadone treatment: a meta-analysis. *Drug Alcohol Depend* 2000;**58**:55–66. doi:10.1016/S0376-8716(99)00068-X

15 Pilling S, Strang J, Gerada C. NICE GUIDELINES: Psychosocial interventions and opioid detoxification for drug misuse: summary of NICE guidance. *Br Med J* 2007;**335**:203–5.

16 Tuten M, Fitzsimons H, Chisolm MS, *et al.* Contingent incentives reduce cigarette

For peer review only

smoking among pregnant, methadone-maintained women: results of an initial feasibility and efficacy randomized clinical trial. *Addiction* 2012;**107**:1868–77. doi:10.1111/j.1360-0443.2012.03923.x

17 Shoptaw S, Rotheram-Fuller E, Yang X, *et al.* Smoking cessation in methadone maintenance. Addiction. 2002;**97**:1317–28.

18 Dunn, K. E, Sigmon, S. C, Thomas, C. S, *et al.* Voucher-based contingent reinforcement of smoking abstinence among methadone-maintained patients: a pilot study. *J Appl Behav Anal* 2008;**41**:527–38. doi:10.1901/jaba.2008.41-527

19 Dunn KE, Sigmon SC, Reimann EF, *et al.* A Contingency-Management Intervention to Promote Initial Smoking Cessation Among Opioid-Maintained Patients. *Exp Clin Psychopharmacol* 2010;**18**:37–50. doi:10.1037/a0018649

20 Sigmon SC, Miller ME, Meyer AC, *et al.* Financial incentives to promote extended smoking abstinence in opioid-maintained patients: A randomized trial. *Addiction* 2016;**111**:903–12. doi:10.1111/add.13264

21 Topp L, Islam MM, Day CA. Relative efficacy of cash versus vouchers in engaging opioid substitution treatment clients in survey-based research. J. Med. Ethics. 2013;**39**:253–6.

22 Vandrey R, Bigelow GE, Stitzer ML. Contingency management in cocaine abusers: a dose-effect comparison of goods-based versus cash-based incentives. *Exp Clin Psychopharmacol* 2007;**15**:338–43. doi:10.1037/1064-1297.15.4.338

23 Festinger DS, Dugosh KL, Kirby KC, *et al.* Contingency management for cocaine treatment: cash vs. vouchers. *J Subst Abuse Treat* 2014;**47**:168–74. doi:10.1016/j.jsat.2014.03.001

24 Viechtbauer W, Smits L, Kotz D, *et al.* A simple formula for the calculation of sample size in pilot studies. *J Clin Epidemiol* 2015;**68**:1375–9. doi:10.1016/j.jclinepi.2015.04.014

25 Sealed Envelope Ltd. Simple randomisation service. 2016.https://www.sealedenvelope.com/simple-randomiser/v1/

26 World Medical Association Declaration of Helsinki: ethical principles for medical research involving human subjects. *JAMA* 2013;**310**:2191–4. doi:10.1001/jama.2013.281053

27 McEwen A. National Centre for Smoking Cessation and Training (NCSCT) Standard Treatment Programme. Second Edition. 2014.

28 National Institute for Health and Care Excellence. Smoking: supporting people to stop. 2012.http://www.nice.org.uk/guidance/qs43/resources/smoking-supporting-people-to-stop-2098665030085 (accessed 29 Jan 2016).

29 E-Burn. Product information - e-burn. http://e-burn.com/product-information/ (accessed 23 Feb 2017).

30 West R, Hajek P, Stead L, *et al.* Outcome criteria in smoking cessation trials: proposal for a common standard. *Addiction* 2005;**100**:299–303. doi:10.1111/j.1360-0443.2004.00995.x

For peer review only - http://bmjopen.bmj.com/site/about/guidelines.xhtml

31 Dobbie F, Hiscock R, Leonardi-Bee J, *et al.* Evaluating Long-term Outcomes of NHS Stop Smoking Services (ELONS): a prospective cohort study. *Health Technol Assess* 2015;**19**:1–156. doi:10.3310/hta19950

32 Thabane L, Ma J, Chu R, *et al.* A tutorial on pilot studies: the what, why and how. *BMC Med Res Methodol* 2010;**10**:1.

33 Senn B, Kirsch M, Sanz CC, *et al.* Developing and evaluating complex interventions: the new Medical Research Council guidance. *Studies* 2013;**59**:587–92.

For peer review only

For peer review only - http://bmjopen.bmj.com/site/about/guidelines.xhtml

Appendix

Appendix 1 Demographic and Smoking behaviour questionnaire

**Basic Demographics**	
Participant ID	
Gender	Male, Female, Not reported
Pregnant	Yes, No, Not reported
Breastfeeding	Yes, No, Not reported
Eligible for free prescriptions?	Yes, No, Not reported
Ethnic Group	White British, White Irish, White Other, Mixed White & Black Caribbean, Mixed White & Black African, Mixed White & Asian, Mixed Other, Asia/Asian Brit – Indian, Asia/Asian Brit – Pakistani, Asia/Asian Brit – Bangladeshi, Asia/Asian Brit – Other, Black/Black Brit - Caribbean<br>Black/Black Brit – African, Black/Black Brit – Other, Chinese, Any other ethnic group
Employment Status	Full time student, Never Worked/Unemployed for over 1 year, Retired, Sick/Disabled/Unable to return to work, Home carer (unpaid), Managerial/Professional, Intermediate occupation (e.g. clerical worker), Routine & Manual occupation (e.g. electrician) Other

For peer review only - http://bmjopen.bmj.com/site/about/guidelines.xhtml

How did you hear about the service?	GP , Practice nurse, Pharmacist, Other Professional, NHS National smoking helpline, Internet, Family/Friends, Previous user of the service, Newspaper or magazine, TV, Poster/leaflet, Other

**Quitting**			
Quitting confidence	1 (Not at all) – 10 (Very)		
Quitting importance	1 (Not at all) – 10 (Very)		
Quitting Readiness	1 (Not at all) – 10 (Very)		
Tried to stop smoking before?	Yes / No	# Times:	
# weeks since last quit attempt			
Longest period of abstinence			
Have you tried NRT?	Yes / No	Types:	
		How long used for	
Ever tried Zyban/Champix?	Yes / No	How long used for	
Have you used other stop smoking aids?	Yes / No	Please Specify	

For peer review only - http://bmjopen.bmj.com/site/about/guidelines.xhtml

Smoking Behaviour	
What type of tobacco do you smoke?	Cigarettes, Roll-ups, Cigars, Oral
How many cig. Do you smoke per day?<br>(if hand rolled, how many ounces per<br>week - 0.5 oz is 12.5g, or 20 cigs)	
How soon after waking do you have your first cig.?	Less than 5 mins, 5-15 mins, 15-30 mins, 30-60 mins, 1-2 hours, More than 2 hours
How many years have you smoked?	
Age started smoking	
Live with a smoker?	

Contact information	
Mobile Phone	
Landline Number	
Email	-
Friend's contact details	-

For peer review only - http://bmjopen.bmj.com/site/about/guidelines.xhtml

Appendix 2 Participant Information Sheet

Study title: Addition of contingency management to stop smoking services for in-treatment opiate addicts: a randomised controlled pilot study

Participant Information Sheet

V1 14/03/2016
IRAS ID: 171709

South London and Maudsley NHS Foundation Trust

KING'S College LONDON Institute of Psychiatry, Psychology & Neuroscience

Smoking Cessation Study

We invite you to take part in a research study

- Before deciding to take part in the study, it is important for you to understand why the research is being done and what taking part will involve
- Please take time to read the following information carefully and discuss it with friends and relatives if you wish
- You are free to decide whether or not to take part in this study. If you choose not to take part this will not affect the care that you receive at Lorraine Hewitt House or anywhere else
- If there is anything that is not clear or you would like more information, then please ask

Important things you need to know

- Tobacco smoking is very common amongst opiate and methadone users. This makes them likely to experience negative health effects.
- Stopping smoking is one of the best things you can do for your health. It can also reduce the discomfort resulting from opiate use treatment.
- Rewards are one way of helping people stop smoking. This is sometimes called contingency management.
- Contingency management has been shown to work well in changing lots of different behaviours. We want to see whether it could help opiate use patients to stop smoking.
- The aim of this study, is to see whether or not it would be possible to test this treatment in a larger trial
- If you take part in the study, you are free to withdraw from the study at any time, **without giving any reason**. If requested, any data that we have collected from you will be destroyed.
- We might ask you to fill out a small questionnaire if you do decide to withdraw from the study, to help us improve our interventions in the future. There is no obligation to complete this questionnaire though.

Requirements

In order to take part in the study you need to:

- Be in treatment for opiate addiction
- Smoke at least 10 cigarettes per day
- Be between 18 and 65 years old
- Must **NOT** be in treatment for any other drug addiction
- Must **NOT** be participating in any other research

What will taking part involve?

- This study is for people who want to stop smoking, and will attend the stop smoking clinic at Lorraine Hewitt house.
- You will need to come to Lorraine Hewitt House **once a week** on a Monday, Wednesday or Friday to attend the smoking clinic. You will have to do this for a total of 6 weeks.
- At the first study visit you will be asked questions about your age and work history etc. You will also be asked to plan to quit smoking for the following week.
- Every time you come into the clinic, you will have to blow into a machine that measures chemicals in your breath. This is how we know if you have been smoking or not.
- You will be put into one of two groups at random (e.g. by coin toss). In one group you can earn rewards for attending the stop smoking clinic and not smoking. In the other group you can earn rewards just for attending the clinic.
- Rewards will be 'Love2Shop' vouchers
- The amount of money that you earn each time you meet the criteria for that group (attending the stop smoking clinic and not smoking in one, and just attending the stop smoking clinic in the other) will start at £5 and will double each time you meet the criteria up to £40. The diagram below shows how much you will earn if you meet the criteria for reward for the duration of the study

	SSS Week/Visit Number						
	1	2	3	4	5	6	
	Intervention Week/Visit Number						
		1	2	3	4	5	**Total**
Reward Value	£0.00	£5.00	£10.00	£20.00	£40.00	£40.00	**£115.00**

- If you don't meet the criteria though you won't get paid for that visit to the clinic, and the amount that you get paid for the next time you do will start again at £5, and will increase each time like before.

Benefits of taking part

- You could earn up to £115 just by attending the stop smoking clinic and stopping smoking
- Taking part may help you to stop smoking for good. This will help improve your general health greatly
- By taking part, you will be helping us to better understand how we can help other people to stop smoking

Possible disadvantages of taking part

- Giving up smoking can result in a number of withdrawal symptoms that may cause discomfort. The behavioural support and nicotine replacement therapy that you will receive as part of the normal smoking cessation clinic is designed to help this.

Frequently asked questions and further information

- **What will happen if I don't want to carry on with the study?** If at any point during the study you decide that you no longer want to take part, you can withdraw without giving any reason. All you need to do is tell anyone at the clinic related with the study that this is the case, and you will immediately be withdrawn from the study. You can also request that all of the data collected from you be destroyed.
- **How will my information be kept confidential?** Any data stored about you will be anonymous, and will not contain any data that would allow you to be identified. All information recorded from you will be held on a secure computer system at King's College London, in an encrypted format that can only be accessed the research team involved with the study.
- **What will happen to the results of this study?** The results of this study will be used by the primary researcher Tom Ainscough as part of his doctoral thesis, will be written up as an academic paper to be published, and will help inform the design of future research.
- **What if I want to know the results of the study when it finishes?** If you want to be informed of the results of the study once it has finished, this information will be made freely available at Lorraine Hewitt House. Just ask at the reception.
- **Who is organising and funding this study?** The study is organised through the Institute of Psychology, Psychiatry and Neuroscience, King's College London and the South London and Maudsley NHS Trust, and is funded by the Medical Research Council (http://www.mrc.ac.uk/)
- **Who has reviewed this study?** The study design has been reviewed by both an NHS ethics committee and the Research and Development department of the Institute of Psychology, Psychiatry and Neuroscience, King's College London
- **Where can I find more information about research?** For more general information about research you can visit either http://www.invo.org.uk/ or www.testingtreatments.org
- **Who can I contact for more information about this study?** If you need any further information about the study, please contact Tom Ainscough by emailing thomas.ainscough@kcl.ac.uk or calling 020 7848 5727

Appendix 3 end of treatment satisfaction survey

Study title: Addition of contingency management to stop smoking services for in-treatment opiate addicts: a randomised controlled pilot study

South London and Maudsley NHS Foundation Trust NHS KING'S College LONDON Institute of Psychiatry, Psychology & Neuroscience

Smoking Study Questionnaire

Participant ID: Date:

Please circle the ONE response you feel most appropriate:

1	**Would you recommend this service to other smokers who want to stop smoking?**	Yes	No	Unsure
2	**In the event that you started smoking again would you go back to the service for help with stopping smoking?**	Yes	No	Unsure
3	**If you returned to the service for help with stopping smoking in the future do you think that you would be welcomed back?**	Yes	No	Unsure
4	**When you contacted the service were you given an appointment date or told how long you would have to wait?**	Yes	No	Unsure
5	**Was the length of time you had to wait for your first appointment acceptable to you?**	Yes	No	Unsure
6	**Are the appointment times you were given convenient for you?**	Yes	No	Unsure
7	**Is the place where you go for your appointments convenient for you to get to?**	Yes	No	Unsure
8	**Was the information that you were given about the choice of medication helpful?**	Yes	No	Unsure
9	**Was it easy to get hold of your medicine once you had chosen which medication you were going to use for your stop smoking attempt?**	Yes	No	Unsure

For peer review only - http://bmjopen.bmj.com/site/about/guidelines.xhtml

Please circle the ONE response you feel most appropriate:

10	Overall, how satisfied are you with the support you have received to stop smoking?					
	Very Unsatisfied	Unsatisfied	Unsure	Satisfied	Very Satisfied	Not Applicable

11	How satisfied are you with how supportive staff have been?					
	Very Unsatisfied	Unsatisfied	Unsure	Satisfied	Very Satisfied	Not Applicable

12	How helpful has the information and advice that staff have given to you during your appointment been?					
	Very Unhelpful	Unhelpful	Unsure	Helpful	Very Helpful	Not Applicable

13	How helpful has the written information that staff have given to you been?					
	Very Unhelpful	Unhelpful	Unsure	Helpful	Very Helpful	Not Applicable

14	Do you find having your carbon monoxide (CO) reading done at every visit helpful?					
	Very Unhelpful	Unhelpful	Unsure	Helpful	Very Helpful	Not Applicable

Please tick ALL appropriate responses:

15	Which of the following types of nicotine replacement therapy did you receive? (Please circle ALL that apply)					
Nicotine patches	Nicotine gum	Nicotine lozenges	Inhalator	Mouth spray	Nasal spray	Oral Strips

16	Which of the following types of nicotine replacement therapy did you find most useful? (Please circle ALL that apply)					
Nicotine patches	Nicotine gum	Nicotine lozenges	Inhalator	Mouth spray	Nasal spray	Oral Strips

17	Did you receive any vouchers during the intervention? If No please go to question 19	
	Yes	No

For peer review only - http://bmjopen.bmj.com/site/about/guidelines.xhtml

18	If yes, how helpful were the vouchers in stopping smoking?					
	Very Unhelpful	Unhelpful	Unsure	Helpful	Very Helpful	Not Applicable

19	Would you have tried to quit smoking if there were no vouchers being offered?	
	Yes	No

20	Did you receive e-cigarettes during the study? If No, please go to question 22	
	Yes	No

21	If yes, how helpful were e-cigarettes for stopping smoking?					
	Very Unhelpful	Unhelpful	Unsure	Helpful	Very Helpful	Not Applicable

22	If you were to do the study again, what would be more likely to make you take part if you got:			
	Vouchers	Free E-cigarettes	Both	Other (please say):

23	Have you smoked since your last appointment with the service?			
	No, not a single puff	Yes, just a few puffs	Yes, 1-5 cigarettes	More Than 5 Cigarettes

For peer review only

For peer review only - http://bmjopen.bmj.com/site/about/guidelines.xhtml

SPIRIT

STANDARD PROTOCOL ITEMS: RECOMMENDATIONS FOR INTERVENTIONAL TRIALS

SPIRIT 2013 Checklist: Recommended items to address in a clinical trial protocol and related documents*

Section/item	Item No	Description	Addressed on page number
**Administrative information**			
Title	1	Descriptive title identifying the study design, population, interventions, and, if applicable, trial acronym	1
Trial registration	2a	Trial identifier and registry name. If not yet registered, name of intended registry	1
	2b	All items from the World Health Organization Trial Registration Data Set	N/A
Protocol version	3	Date and version identifier	
Funding	4	Sources and types of financial, material, and other support	9
Roles and responsibilities	5a	Names, affiliations, and roles of protocol contributors	1, 9
	5b	Name and contact information for the trial sponsor	9
	5c	Role of study sponsor and funders, if any, in study design; collection, management, analysis, and interpretation of data; writing of the report; and the decision to submit the report for publication, including whether they will have ultimate authority over any of these activities	10
	5d	Composition, roles, and responsibilities of the coordinating centre, steering committee, endpoint adjudication committee, data management team, and other individuals or groups overseeing the trial, if applicable (see Item 21a for data monitoring committee)	N/A

For peer review only - http://bmjopen.bmj.com/site/about/guidelines.xhtml

**Introduction**			
Background and rationale	6a	Description of research question and justification for undertaking the trial, including summary of relevant studies (published and unpublished) examining benefits and harms for each intervention	1,2
	6b	Explanation for choice of comparators	1-4
Objectives	7	Specific objectives or hypotheses	3
Trial design	8	Description of trial design including type of trial (eg, parallel group, crossover, factorial, single group), allocation ratio, and framework (eg, superiority, equivalence, noninferiority, exploratory)	3-7
**Methods: Participants, interventions, and outcomes**			
Study setting	9	Description of study settings (eg, community clinic, academic hospital) and list of countries where data will be collected. Reference to where list of study sites can be obtained	5-6
Eligibility criteria	10	Inclusion and exclusion criteria for participants. If applicable, eligibility criteria for study centres and individuals who will perform the interventions (eg, surgeons, psychotherapists)	3
Interventions	11a	Interventions for each group with sufficient detail to allow replication, including how and when they will be administered	5-7
	11b	Criteria for discontinuing or modifying allocated interventions for a given trial participant (eg, drug dose change in response to harms, participant request, or improving/worsening disease)	2
	11c	Strategies to improve adherence to intervention protocols, and any procedures for monitoring adherence (eg, drug tablet return, laboratory tests)	N/A
	11d	Relevant concomitant care and interventions that are permitted or prohibited during the trial	6
Outcomes	12	Primary, secondary, and other outcomes, including the specific measurement variable (eg, systolic blood pressure), analysis metric (eg, change from baseline, final value, time to event), method of aggregation (eg, median, proportion), and time point for each outcome. Explanation of the clinical relevance of chosen efficacy and harm outcomes is strongly recommended	6
Participant timeline	13	Time schedule of enrolment, interventions (including any run-ins and washouts), assessments, and visits for participants. A schematic diagram is highly recommended (see Figure)	5

For peer review only - http://bmjopen.bmj.com/site/about/guidelines.xhtml

Sample size	14	Estimated number of participants needed to achieve study objectives and how it was determined, including clinical and statistical assumptions supporting any sample size calculations	______5_______
Recruitment	15	Strategies for achieving adequate participant enrolment to reach target sample size	______5_______
**Methods: Assignment of interventions (for controlled trials)**			
Allocation:			
Sequence generation	16a	Method of generating the allocation sequence (eg, computer-generated random numbers), and list of any factors for stratification. To reduce predictability of a random sequence, details of any planned restriction (eg, blocking) should be provided in a separate document that is unavailable to those who enrol participants or assign interventions	______4_______
Allocation concealment mechanism	16b	Mechanism of implementing the allocation sequence (eg, central telephone; sequentially numbered, opaque, sealed envelopes), describing any steps to conceal the sequence until interventions are assigned	______4_______
Implementation	16c	Who will generate the allocation sequence, who will enrol participants, and who will assign participants to interventions	______4_______
Blinding (masking)	17a	Who will be blinded after assignment to interventions (eg, trial participants, care providers, outcome assessors, data analysts), and how	_____ 4______
	17b	If blinded, circumstances under which unblinding is permissible, and procedure for revealing a participant's allocated intervention during the trial	_____N/A______
**Methods: Data collection, management, and analysis**			
Data collection methods	18a	Plans for assessment and collection of outcome, baseline, and other trial data, including any related processes to promote data quality (eg, duplicate measurements, training of assessors) and a description of study instruments (eg, questionnaires, laboratory tests) along with their reliability and validity, if known. Reference to where data collection forms can be found, if not in the protocol	______6-7______
	18b	Plans to promote participant retention and complete follow-up, including list of any outcome data to be collected for participants who discontinue or deviate from intervention protocols	______8______

Data management	19	Plans for data entry, coding, security, and storage, including any related processes to promote data quality (eg, double data entry; range checks for data values). Reference to where details of data management procedures can be found, if not in the protocol	______N/A______
Statistical methods	20a	Statistical methods for analysing primary and secondary outcomes. Reference to where other details of the statistical analysis plan can be found, if not in the protocol	______8_______
	20b	Methods for any additional analyses (eg, subgroup and adjusted analyses)	______N/A______
	20c	Definition of analysis population relating to protocol non-adherence (eg, as randomised analysis), and any statistical methods to handle missing data (eg, multiple imputation)	_______6__ _____
**Methods: Monitoring**			
Data monitoring	21a	Composition of data monitoring committee (DMC); summary of its role and reporting structure; statement of whether it is independent from the sponsor and competing interests; and reference to where further details about its charter can be found, if not in the protocol. Alternatively, an explanation of why a DMC is not needed	______N/A _____
	21b	Description of any interim analyses and stopping guidelines, including who will have access to these interim results and make the final decision to terminate the trial	______N/A _____
Harms	22	Plans for collecting, assessing, reporting, and managing solicited and spontaneously reported adverse events and other unintended effects of trial interventions or trial conduct	______N/A _____
Auditing	23	Frequency and procedures for auditing trial conduct, if any, and whether the process will be independent from investigators and the sponsor	_____________
**Ethics and dissemination**			
Research ethics approval	24	Plans for seeking research ethics committee/institutional review board (REC/IRB) approval	______9_______
Protocol amendments	25	Plans for communicating important protocol modifications (eg, changes to eligibility criteria, outcomes, analyses) to relevant parties (eg, investigators, REC/IRBs, trial participants, trial registries, journals, regulators)	______N/A______

For peer review only - http://bmjopen.bmj.com/site/about/guidelines.xhtml

Consent or assent	26a	Who will obtain informed consent or assent from potential trial participants or authorised surrogates, and how (see Item 32)	_____3______
	26b	Additional consent provisions for collection and use of participant data and biological specimens in ancillary studies, if applicable	______N/A _____
Confidentiality	27	How personal information about potential and enrolled participants will be collected, shared, and maintained in order to protect confidentiality before, during, and after the trial	______7_______
Declaration of interests	28	Financial and other competing interests for principal investigators for the overall trial and each study site	_____9-10_____
Access to data	29	Statement of who will have access to the final trial dataset, and disclosure of contractual agreements that limit such access for investigators	______9_______
Ancillary and post-trial care	30	Provisions, if any, for ancillary and post-trial care, and for compensation to those who suffer harm from trial participation	______N/A_____
Dissemination policy	31a	Plans for investigators and sponsor to communicate trial results to participants, healthcare professionals, the public, and other relevant groups (eg, via publication, reporting in results databases, or other data sharing arrangements), including any publication restrictions	_____________
	31b	Authorship eligibility guidelines and any intended use of professional writers	______N/A_____
	31c	Plans, if any, for granting public access to the full protocol, participant-level dataset, and statistical code	____________
**Appendices**			
Informed consent materials	32	Model consent form and other related documentation given to participants and authorised surrogates	_____________
Biological specimens	33	Plans for collection, laboratory evaluation, and storage of biological specimens for genetic or molecular analysis in the current trial and for future use in ancillary studies, if applicable	______N/A______

*It is strongly recommended that this checklist be read in conjunction with the SPIRIT 2013 Explanation & Elaboration for important clarification on the items. Amendments to the protocol should be tracked and dated. The SPIRIT checklist is copyrighted by the SPIRIT Group under the Creative Commons "Attribution-NonCommercial-NoDerivs 3.0 Unported" license.

For peer review only - http://bmjopen.bmj.com/site/about/guidelines.xhtml

Figure**.** Example template of recommended content for the schedule of enrolment, interventions, and assessments.*

	Enrolment		Post-allocation					Post study	Follow-up
**TIMEPOINT****	*-t_1*	*Week 1*	*Week 2*	*Week 3*	*Week 4*	*Week 5*	*Week 6*		*6 Months*
**ENROLMENT:**									
**Eligibility screen**	X								
**Informed consent**	X								
Demographics collection	X								
**Allocation to condition**		X							
**INTERVENTIONS:**									
CM Smoking abstinence			◆	—	—	—	◆		
CM Clinic attendance			◆	—	—	—	◆		
**ASSESSMENTS:**									
**Numbers completing treatment**							X		
Demographics	X								
Breath CO			◆	—	—	—	◆		X
Point prevalence smoking			◆	—	—	—	◆		X
Opiate treatment information								X	
Illicit drug use								X	

*Recommended content can be displayed using various schematic formats. See SPIRIT 2013 Explanation and Elaboration for examples from protocols.

**List specific timepoints in this row.

For peer review only
